# Determination of some B Vitamins in Sour Cherry Juice Using Dispersive Liquid-liquid Microextraction Followed by High-performance Liquid Chromatography

**Published:** 2014

**Authors:** Parvin Parsaei, Manouchehr Bahmaei, AliReza Ghannadi

**Affiliations:** a *Department of Chemistry, Islamic Azad University, North Tehran Branch, Tehran, Iran. *; b *School of Pharmacy and Pharmaceutical Sciences and Isfahan Pharmaceutical Research Center, Isfahan University of Medical Sciences, Isfahan, Iran.*

**Keywords:** Dispersive liquid-liquid microextraction (DLLME), High performance liquid chromatography-ultraviolet detector (HPLC-UV), Thiamine, Nicotinamide, Pyridoxine

## Abstract

Dispersive liquid-liquid microextraction method (DLLME) combined with high-performance liquid chromatography-ultraviolet detection (HPLC-UV) was used to determine thiamine (B_1_), nicotinamide (B_3_) and pyridoxine (B_6_) in sour cherry juice. This method was rapid, simple and sensitive. Separation was accomplished using a C_18_ column. The optimum chromatographic conditions were found to be: mobile phase consisted of 8% methanol and 92% aqueous phase (1% (V/V) acetic acid water solution); flow rate, 0.7 mL/min; detection wavelength, 260 nm and pH, 3.3. The extraction efficiency of thiamine, nicotinamide and pyridoxine was influenced by factors such as: additional salt effect, the kind and volume of disperser and extraction solvents. In this research, the limit of detection (LOD) and quantification (LOQ) were 0.9 and 3 ng/mL for thiamine, 1.5 and 5 ng/mL for nicotinamide, 0.9 and 3 ng/mL for pyridoxine. The relative standard deviations (RSDs) were less than 2.87% (n=3). An appropriate linear behavior over the observed concentration range was obtained with the value of R²>0.996 for the target vitamins. This method was successfully applied to the sour cherry juice samples. Sour cherry var. Gise (Prunus cerasus var. Gise), which was used in this research, was a local variety of the sour cherry with large stone, double flowers, double fruits, dark red skin and dark red juice. This variety was identified in high altitude areas of Isfahan province after five years of study, since 2005, by Agricultural and Natural Resources Research Center of Isfahan.

## Introduction

B vitamins exist in an extended range of foods. They are water-soluble vitamins and it was once believed that all of them were single vitamin. Thiamin is necessary for the synthesis of neurotransmitters such as gama-aminobutyric acid (GABA) and acetylcholine (1). It is a cofactor involved in amino acid catabolism and carbohydrate catabolism (2). Coenzymes, which are involved in metabolic functions such as nicotinamide adenine dinucleotide hydrogen (NADH) and nicotinamide adenine dinucleotide (NAD+), have nicotinamide as a precursor (3). The active form of pyridoxine is pyridoxal-5-phosphate. Several neurotransmitters need pyridoxal-5-phosphate for synthesis. It is an important cofactor that changes tryptophan to serotonin (4). 

Sour cherry has important compounds such as: anthocyanins, catechins, flavonal glycosides, melatonin, hydroxycinnamates, chlorogenic acid, cyanidin, B and C vitamins (5, 6). Scientists found the efficacy of cherries in reducing muscle pain during running (7), preventing symptoms of muscle damage (8), reducing muscle damage caused by intensive strength exercise (9), decreasing oxidative stress (10) and influencing neuronal cells (11).They also recognized that the cherries compounds are anti-gout (12) and anti-inflammatory (13).

Now, the researches focus on techniques that need small amounts of solvents and samples. The new techniques such as Solid phase microextraction (SPME) (14, 15) and Liquid phase microextraction (LPME) (16, 17, 18) need small amounts of samples, but they have long extraction times. To minimize the extraction time and the amount of samples and solvents, Rezaea *et al*., innovated Dispersive liquid liquid microextraction (DLLME) (19). DLLME technique uses two solvents: an organic solvent insoluble in water, called extraction solvent, and a water soluble solvent called disperser solvent.

A wide range of analytical separation methods have been performed on reversed phase chromatography as discussed by Melander and Horvath in 1980 (20). The factors that influence the selection of a suitable column for the separation are retention times, efficiency, tailing factors, the appropriate temperature for the analytes and its proper pH (21). Separation was accomplished using a C_18_ column, thus the Reversed-phase HPLC was performed.

The aim of this study was to quantify the amount of vitamin B_1_ (thiamine), vitamin B_3_ (nicotinamide) and vitamin B_6_ (pyridoxine) from sour cherry juice using dispersive liquid-liquid microextraction followed by HPLC. 

## Experimental

***Chemical and reagents***

Analytical grade reagents (99.8% acetic acid, 99.9% ethanol and chloroform), HPLC-grade solvents (99.9% methanol and 99.9% acetonitril) were purchased from Merck, Germany. Hexane 1-sulfonic acid sodium salts for ion pair chromatography and sodium chloride were purchased from Merck, Germany. Authentic standards, 99.6% thiamine hydrochloride (vitamin B_1_), 99.7% nicotinamide (vitamin B_3_) and 99% pyridoxine hydrochloride (vitamin B_6_) were purchased from Amin pharmaceutical Co. (Isfahan, Iran). We produced de-ionized water using a Milli-Q reverse osmosis purification system.

The preliminary standards of target vitamins were formed by weighting out 1.00 mg of each vitamin into a 10 mL volume flask. By adding ultra pure water, the volumes of standard samples were brought up to 10 mL.

***Instrumentation***

The HPLC system (Waters, Milford, MA, USA) consisted of Waters 2487 dual λ absorbance detector. The volume of injection loop was 10.0 µL. Separation was carried out on HS-OSD C_18_ column (25 cm×4.6 mm with 5 µm particle size) from Shinwa (Japan). We optimized the chromatographic conditions to determine vitamins B_1_, B_3_ and B_6_. We found that the mobile phase consisting of 8% methanol and 92% aqueous phase (1% (V/V) acetic acid water solution) had the best resolution factors, the satisfactory retention times and the sharpest peaks for vitamins B_1_, B_3_ and B_6_, thus, we used these conditions. The pH of the mobile phase was 3.3. The flow rate of the mobile phase was adjusted at 0.7 mL/min. The wavelength of the detector was optimized at 260 nm and the isocratic elution method was used (22). For centrifuging, we used a Hettich centrifuge (D-7200, Germany). A pH meter from metrohm (Switzerland) was used. The syringes volumes, 1.0 mL and 100 µL were purchased from Hamilton Co (USA).

***Standard solution and calibration curves***

Stock solutions (100 ng/mL) of thiamine, nicotinamide and pyridoxine were prepared by dissolving 1 mg of their solids in 10 mL de-ionized water in a calibrated flask. The temperature was adjusted at 4 °C. We diluted the stock solutions to make working solutions. The external standard method was used for quantitative analysis. The appropriate amounts of the stock solutions were added to the sour cherry juice to prepare 

sour cherry juice standards. The calibration curves were achieved based on the concentrations of target vitamins and the peak areas of them. The analytes concentrations were calculated based on the calibration curves. 

***Preparation of sour cherry juice***

Sour cherry var. Gise (Prunus cerasus var. Gise) was identified and specified by Agricultural and Natural Resources Research Center of Isfahan.

We filtered the sour cherry juice with Whatman filter paper no.40. Aliquot of 10 mL from this juice was placed in graduated centrifuge test tubes. These solutions were centrifuged for 10 min at 4500 rpm and the upper part was taken with a syringe and then filtered by 0.22 µm syringe filters (23). The sour cherries, stored in 4 ºC at the dark place, were stable for 4 days, and the sour cherry juice was daily prepared.

***Dispersive liquid-liquid microextraction procedure***

For DLLME, the volume of 1.00 mL of the Sour cherry juice was poured in a 10 mL conical bottomed glass test tube; then, the injection of 0.5 mL methanol (disperser solvent) and 0.4 mL chloroform (extraction solvent) to the sample was performed rapidly by a 1 mL Hamilton syringe. When the chloroform was dispersed, the cloudy solution was obtained from it. Then, in few minutes, a fine droplet of chloroform was extracted. The glass test tube was centrifuged for 10 min at the rate of 4500 rpm. The sedimented phase was performed at the bottom of test tube, which was the fine droplet of chloroform extracting the analyte. Then the sedimented phase was separated using a 1 mL Hamilton syringe. It was poured to the small vial. The small vial content was placed under the nitrogen flow. When the vial content was dried, it was dissolved in 0.2 mL of methanol, and a 0.22 µm PTFE syringe filter was used to filter it (23).

## Result and Discussion

***Adjusting HPLC-UV system***

**Figure 1:**
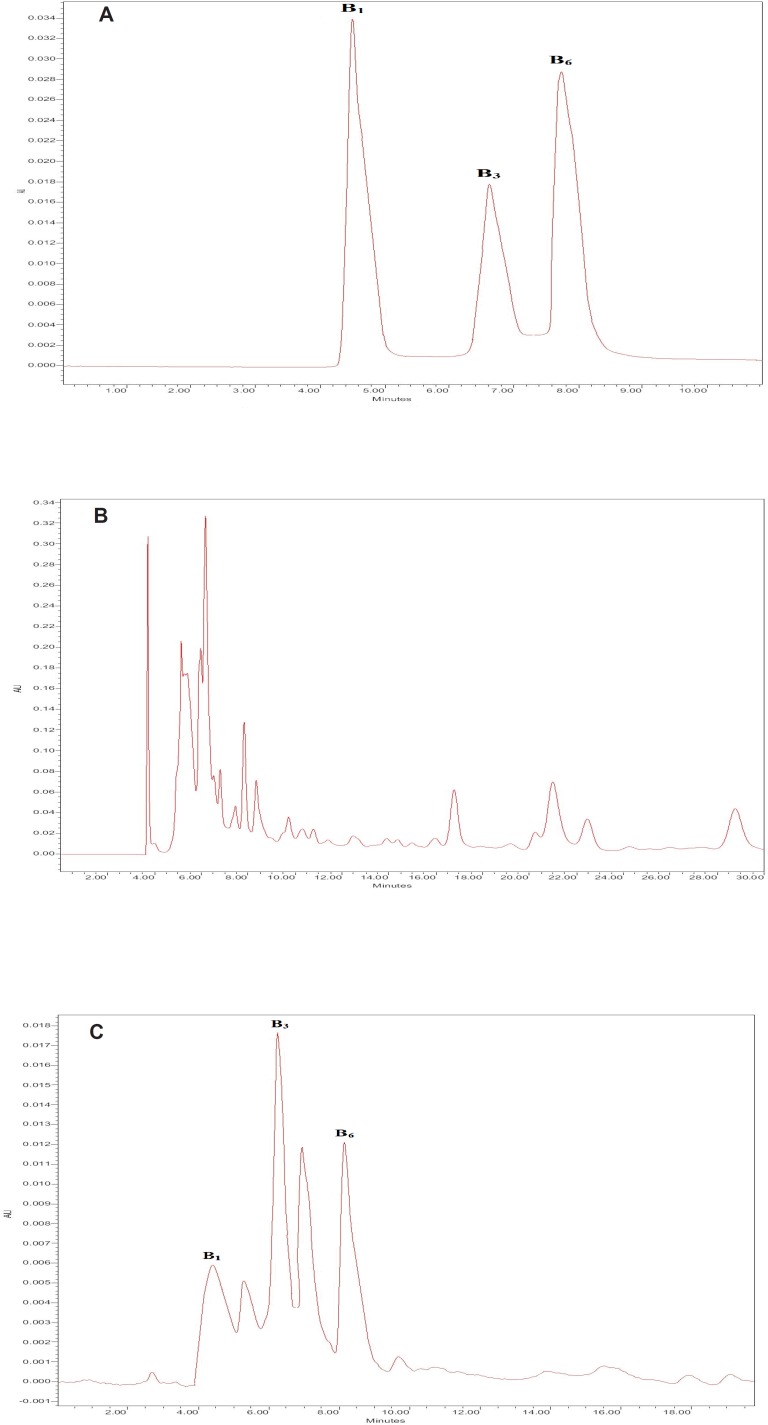
The HPLC chromatograms of thiamine (B_1_), nicotinamide (B_3_) and pyridoxine (B_6_) for standard solutions. A: After extraction using DLLME; B: sour cherry juice without extraction; C: sour cherry juice after extraction using DLLME.

The maximum UV spectra absorption of thiamine, nicotinamide and pyridoxine were 260 nm, therefore, the HPLC wavelength was adjusted at 260 nm. We used a C_18_ column and examined different concentrations of the aqueous phase (acetic acid water solution) and methanol. The optimum conditions were obtained to be: mobile phase consisted of 8% methanol and 92% aqueous phase (1 % (V/V) acetic acid water solution); pH 3.3. It had the sharpest peaks and the best resolution factors for thiamine, nicotinamide and pyridoxine. Figure 1A shows the HPLC chromatograms of thiamine, nicotinamide and pyridoxine for standard solutions after DLLME. The retention times of target vitamins were 4.6, 6.6 and 8.0 min for thiamine, nicotinamide and pyridoxine, respectively.

***DLLME method Optimization***

In this research, the type and the volume of extraction and disperser solvents, and the salt effect were studied and optimized.

***Type of disperser solvent***

The disperser solvent should be miscible in the extraction solvent and sample solution.

**Figure 2:**
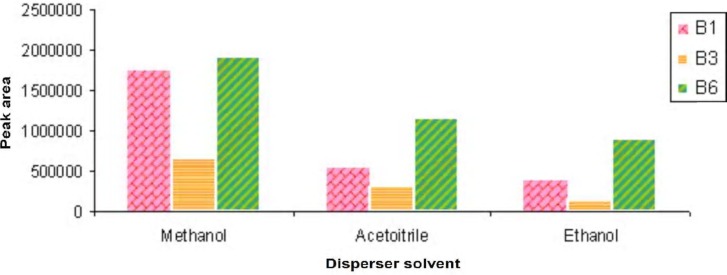
The comparative influence of type of disperser solvent on DLLME of B vitamins.

To select the best disperser solvent in this research, we studied the effect of methanol, ethanol and acetonitrile as disperser solvents. We found that methanol could be used well as disperser solvent because of the maximum peak areas (Figure 2). 

***The comparative influence of disperser solvent volume on DLLME***

**Figure 3:**
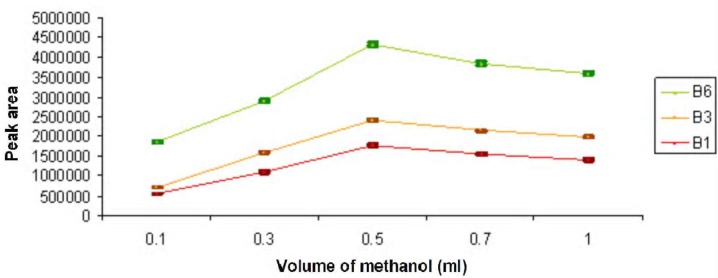
The comparative influence of volume of disperser solvent on DLLME of B vitamins.

To study the disperser solvent volume on DLLME, the volume of extraction solvent was invariable (chloroform, 0.4 mL), but the volumes of methanol were variable from 0.1 mL to 1 mL (0.1, 0.3, 0.5, 0.7, 1.00 mL). The optimum volume of disperser solvent used in this research was 0.5 mL. It was observed that firstly, the extraction efficiency was increased when the methanol volume was increased and then decreased. This was because of the decrease in the analyte partition with extraction droplets (Figure 3).

***Type of extraction solvent***

The extraction solvent should have some characteristics: it should be immiscible in water and its density must be higher than that of water. In the present study, chloroform (density1.48 ng/mL) was appropriate to be used as the extraction solvent.

***The comparative influence of extraction solvent volume on DLLME***

**Figure 4:**
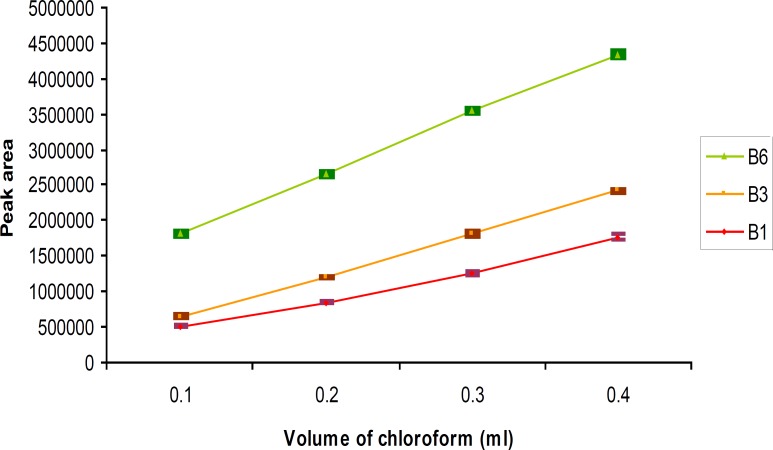
The comparative influence of extraction solvent (chloroform) volume on extraction efficiency

To study the extraction solvent volume on DLLME, the volume of disperser solvent was invariable (methanol, 0.5 mL), but the volume of chloroform was variable from 0.1 mL to 0.4 mL (0.1, 0.2, 0.3, 0.4 mL). It was observed that the chromatogram peak areas were increased when the chloroform volumes were increased (Figure 4).

***The comparative influence of additional salt on DLLME***

**Figure 5:**
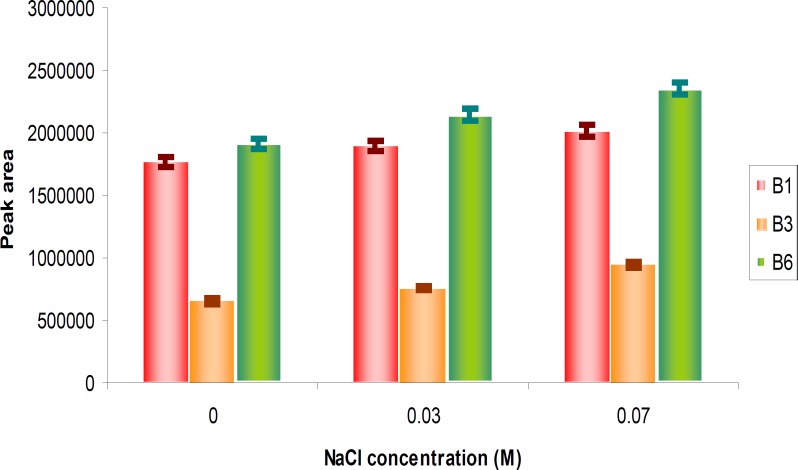
The comparative influence of additional salt on DLLME.

To study the effect of salt on DLLME, we added different concentrations of NaCl from 0-0.07 M to the mixture. The chromatogram peak areas were increased with the increase in NaCl concentration, but salt caused some intrusive peaks in the chromatogram (Figure 5); thus, we did not add any NaCl under the optimum condition. 

***Extraction time Effect***

The time between injecting extraction solvent and starting centrifuging the samples was named the extraction time. In this research, we studied the effect of it on the extraction efficiency. The extraction time was variable from 1 min to 6 min, but all results of this study were constant in 1, 2, 3, 4, 5 and 6 min. We understood that extraction time did not have any effect on extraction efficiency. 

***The effect of sour cherry juice matrix***

Determination of thiamine, nicotinamide and pyridoxine in sour cherry juice samples with HPLC was very difficult because of their matrix effect. Diluting the sour cherry juice samples to decrease the matrix effect decreased the analytical signals of analytes. Figure 1B illustrates the sour cherry juice without extraction. Although we observed high interferences in this chromatogram, we found that dispersive liquid liquid microextraction method, shown in Figure 1C removed, all matrix interferences, leading to the best determination of the target vitamins. So, this method was used in this research.

*Recovery test*

The recovery tests of samples extracted with DLLME method with six levels of concentrations are shown in Table 1. The average recoveries (%) for thiamine, nicotinamide and pyridoxine were 102.8, 103.5 and 103.7, respectively.

**Table 1:** Analytical results of thiamine, nicotinamide and pyridoxine in sour cherry juice samples (mean ± SD, n=6).

**Analyte**	**Added**	**Found (ng/mL)**	**Recovery (%)**	**Average recovery** **(%)**
**Thiamine**	0 10 30 50 70 100	0 11.73 ± 2.04 28.35 ± 1.16 50.42 ± 0.35 71.72 ± 2.18 98.90 ± 1.23	- 117.3% 94.5% 100.8% 102.5% 98.9%	102.8%
**Nicotinamide**	0 10 30 50 70 100	0 10.66 ± 0.54 32.68 ± 1.46 51.28 ± 0.97 70.91 ± 1.64 97.85 ± 1.27	- 106.6% 108.9% 102.6% 101.3% 97.9%	103.5%
**Pyridoxine**	0 10 30 50 70 100	0 11.44 ± 0.17 30.53 ± 0.79 51.20 ± 1.10 71.32 ± 1.86 98.15 ± 2.38	- 114.4% 101.8% 102.4% 101.9% 98.2%	103.7%

***Method validation***

The linearity of the method and its sensitivity are described in Table 2. The limit of detection (LOD) LOD=3.3σ/S, and the limit of quantification (LOQ) LOQ=10σ/S were calculated (24). The standard variation was σ (n=6). The relative standard deviations (RSDs) were obtained for the method repeatability and reproducibility. The RSD for the method repeatability was obtained with determination of the sour cherry juice samples at three replicates and six levels of analytes (0, 0.01, 0.03, 0.05, 0.07, 0.1 µg/mL) on one day. The RSD for the method reproducibility was obtained with determination of the sour cherry juice samples at three replicates and six levels of analytes (0, 0.01, 0.03, 0.05, 0.07, 0.1 µg/mL) on three days and three times a day. Typical chromatograms of the blank sour cherry juice and spiked sour cherry juice samples (after DLLME) are given in Figure1. 

**Table 2:** Analytical performance of HPLC-UV of thiamine, nicotinamide and pyridoxine on the C_18_ column.

**Analyte**	**Calibration curve**	**R** ^2^	**Linear range** **(µg/mL)**	**RSD (%)** **Repeatability**	**RSD (%)** **Reproducibility**	**LOD** **(ng/mL)**	**LOQ** **(ng/mL)**
Thiamine	y=812.68x+925.56	0.9984	0.003-0.140	2.31	2.87	0.9	3
Nicotinamide	y=1562.5x+5293.7	0.9963	0.005-0.100	2.85	2.86	1.5	5
Pyridoxine	y=1065.3x+2865.3	0.9977	0.003-0.150	2.12	2.50	0.9	3

***Application***

A wide range of different methods have been used for the determination of B vitamins in natural and pharmaceutical compounds, for instance liquid chromatography with diode-array detection (25, 26, 27), liquid chromatography with mass spectroscopy (26, 28), high performance liquid chromatography with ultraviolet detection (22, 29, 30) and *etc*. However, some of these methods have disadvantages such as time consuming, insensitivity, low enrichment factor, difficult operation, matrix effect, low accuracy, toxic and expensive solvents and *etc*. To overcome these limitations in the determination and extraction of B vitamins DLLME-HPLC-UV is the best option. The accuracy of this method has been validated by using the recovery tests.

Determining the concentrations of thiamine, nicotinamide and pyridoxine in sour cherry juice samples was the aim of this method. The results are shown in Table1.

For the spiked sour cherry juice, samples had the valuable recovery ranges of 94.5%-117.3%. The concentrations of thiamine, nicotinamide and pyridoxine in the sour cherry juice samples were obtained to be 68.9 ± 1.9, 531.5 ± 5.9 and 77.3 ± 0.8 ng/mL, respectively. To examine the method validation, 70 ng/mL of each of the target vitamins were spiked into the sour cherry juice samples and the concentrations obtained in the spiked sour cherry juice samples were found to be 140.2 ± 4.4, 603.3 ± 14.2 and 148.5 ± 3.9 ng/mL, respectively. These values illustrate the sum of B vitamins existing in the sour cherry juice and 70 ng/mL of each of the target vitamins added to the samples. Therefore, the obtained data showed satisfactory recoveries. The chromatograms of sour cherry juice after extraction using DLLME are given in Figure 1C. This cleanup method worked well and produced the sharpest peaks with the least interference. 

## Conclusion

The novel DLLME-HPLC-UV method was evaluated for the determination of thiamine, nicotinamide and pyridoxine in sour cherry juice sample. The method showed advantages when compared with other conventional methods such as short extraction time, the absence of matrix effects and produced sharper peaks with less interference. The relative recoveries of those compounds studied in sour cherry juice were from 94.5% to 117.3%. DLLME method was rapid, simple and reproducible with a high linearity.
